# When Meals Turn Into a Medical Mystery: A Case Report of Sigmoid Colon Impaction by a Chicken Bone

**DOI:** 10.7759/cureus.45955

**Published:** 2023-09-25

**Authors:** Haider Ghazanfar, Abeer Qasim, Dongmin Shin, Haozhe Sun, Ariyo Ihimoyan

**Affiliations:** 1 Internal Medicine, Bronxcare Health System, Bronx, USA; 2 Internal Medicine, BronxCare Health System, New York, USA; 3 Internal Medicine, BronxCare Health System, Bronx, USA; 4 Medicine/Internal Medicine, BronxCare Health System, Bronx, USA; 5 Gastroenterology, BronxCare Health System, Bronx, USA

**Keywords:** sigmoid colon obstruction due to chicken bone, intestinal perforation, foreign body ingestion, gastrointestinal obstruction, chicken bone-induced sigmoid colon impaction

## Abstract

The presence of foreign objects in the digestive system can lead to various complications within the gastrointestinal (GI) tract. In certain cases, ingesting foreign objects can pose a significant dilemma for surgical teams, primarily because they can lead to blockages or punctures in the upper or lower sections of the GI tract. On occasion, foreign objects located in the lower regions of the digestive tract, such as the colon and rectum, might have entered via the anal pathway, thereby carrying the potential risk of causing perforations in the rectum or sigmoid colon. The other complications encompass the creation of abscesses, blockages in the bowel, fistula, and bleeding. Although these issues associated with foreign objects can arise in a healthy bowel, they can pose challenges in diagnosis when occurring in a bowel that is already affected by inflammation, constrictions, or malignancy. We present a unique case report of a 72-year-old female who presented to the emergency department with left lower quadrant pain associated with nausea and vomiting. Following a detailed clinical evaluation and radiographic imaging, a chicken bone was identified as the culprit, causing an unusual impaction in the sigmoid colon. The patient's medical history revealed no prior GI complications, making this case particularly noteworthy. Timely identification and precise diagnosis of complications arising from foreign bodies are essential to efficiently handle and prevent unfavorable consequences.

## Introduction

Ingesting a foreign object ranks among the prevalent causes of seeking care in the emergency ward. The symptoms resulting from such foreign entities within the gastrointestinal (GI) system can range from the complete absence of symptoms in instances where the foreign body is situated in the digestive tract to the emergence of diverse and unspecific indications. Approximately 80% of foreign items traverse the GI tract autonomously, requiring no external intervention. Meanwhile, 20% of cases necessitate endoscopic measures, and a mere 1% necessitate surgical procedures. Among adults, the most commonly swallowed foreign materials include fish bones (constituting 9%-45%), bones (comprising 8%-40%), and dentures (accounting for 4%-18%) [1]. Approximately 20% of swallowed foreign objects do not successfully traverse the GI tract. Foreign objects typically manifest with abdominal discomfort that lacks specific characteristics. While a significant portion of these cases necessitate surgical intervention for resolution, there are instances when endoscopic methods can be employed to retrieve these objects [2]. The rectosigmoid junction is one of the narrowest segments within the GI system. This characteristic makes it a prime candidate for potential complications arising from ingested foreign objects [3]. We present a unique case of a 72-year-old woman who arrived with an obstructed chicken bone in her sigmoid colon. This situation led to an abscess, which was effectively managed through a combination of antibiotics, drainage, and sigmoidoscopy. 

## Case presentation

A 72-year-old female with a medical history of hypertension presented to the emergency room with left lower quadrant (LLQ) abdominal pain associated with nausea and vomiting for three days. Regarding pain, it was non-radiating, sharp, and stabbing in nature and was worse with eating food. She denied experiencing any similar prior episodes. The last bowel movement was a few hours before the presentation, and she described it as a brown stool. The patient has had occasional constipation; however, denied fever, chills, melena, hematochezia, hematemesis, diarrhea, or weight loss. The patient also confirmed not having consumed any foreign object intentionally or inserted one through the rectum. Nevertheless, it's worth noting that the individual had consumed chicken three to four hours before the symptoms began. She denies any surgical procedures except for a tubal ligation performed a long time ago. Additionally, there was no record of any previous endoscopic examinations or any family history of GI malignancy. 

In the emergency room, the patient was hemodynamically stable with a blood pressure of 128/58 mmHg, heart rate of 87 beats/minute, respiratory rate of 18 breaths/minute, saturating 95% on room air, and was afebrile with a body temperature of 98.7 °F. Her physical examination finding was significant for LLQ tenderness without muscle guarding or rebound tenderness. The rest of the examination findings were unremarkable. 

Initial laboratory test revealed leukocytosis (white blood cell [WBC] 11.7 K/µL) with an elevated inflammatory marker level (C-reactive protein [CRP] level of 97.02 mg/L; Table 1).

**Table 1:** Initial lab results.

Labs	Results	Reference range
White blood cell	11.7	4.8-10.8 K/µL
Hemoglobin	12.0	12.0-16.0 g/dL
Hematocrit	37.8	42.0%-51.0%
Platelet	380	150-400 K/µL
Sodium	137	135-145 mEq/L
Potassium	4.5	3.5-5.0 mEq/L
Chloride	96	98-108 mEq/L
Bicarbonate	31	24-30 mEq/L
Glucose	91	70-120 mg/dL
Blood urea nitrogen	10.0	6-20 mg/dL
Creatinine	0.8	0.5-1.5 mg/dL
Calcium	9.5	8.5-10.5 mg/dL
Total protein	7.9	5.8-8.3 g/L
Albumin	4.4	3.2-4.6 g/dL

**Figure 1:**
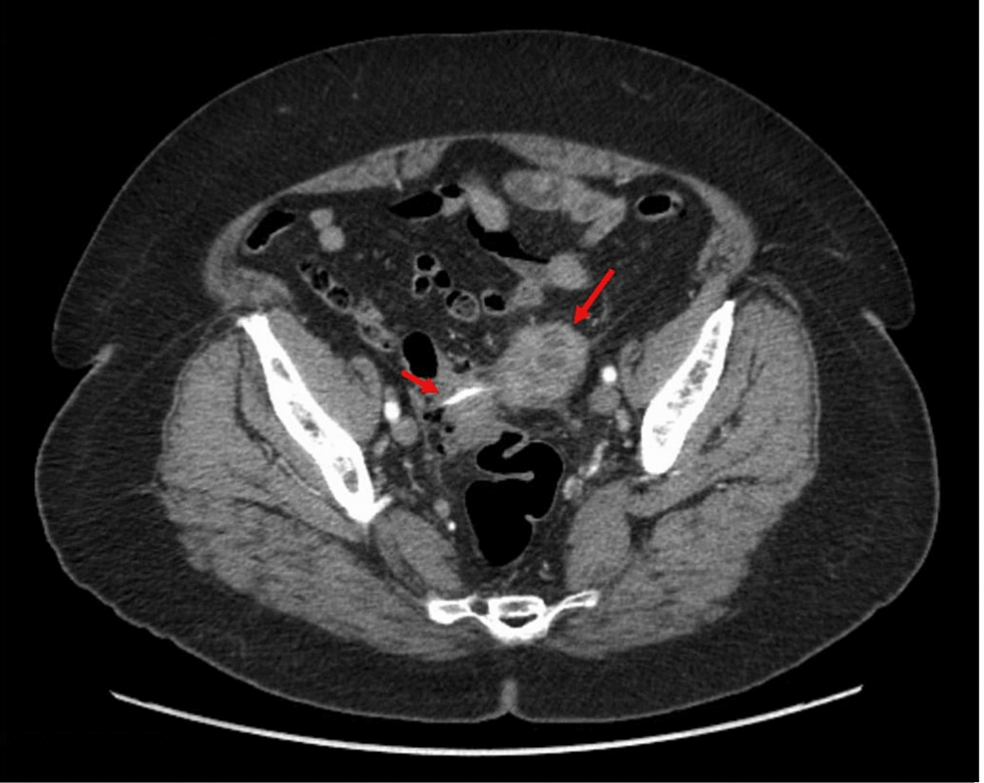
Initial CT abdomen pelvis with contrast image showing a needle-like foreign body with adjacent abdominal abscess measuring 5.0 cm × 4.3 cm × 4.4 cm, as indicated by the arrows. CT, computed tomography

**Figure 2:**
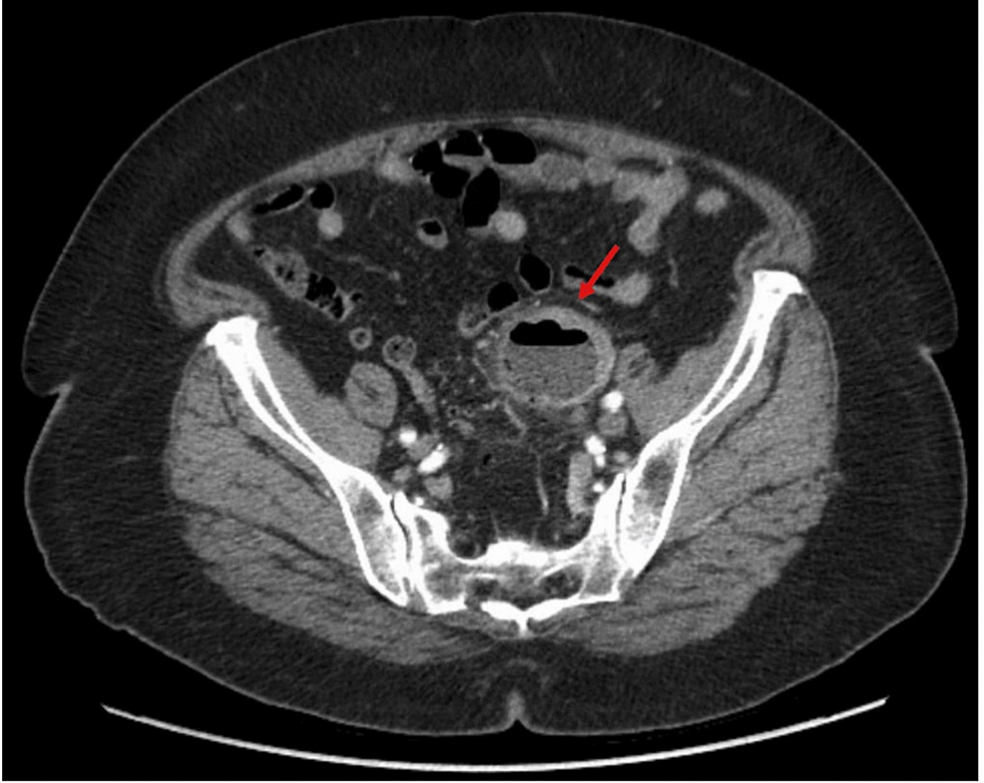
Initial CT abdomen pelvis with contrast image showing a needle-like foreign body with adjacent abdominal abscess measuring 5.0 cm × 4.3 cm × 4.4 cm. CT, computed tomography

She underwent computed tomography (CT) of the abdomen and pelvis with contrast, which revealed a needle-like foreign body in the sigmoid colon, which has perforated the colon with an adjacent abscess measuring 5.0 cm × 4.3 cm × 4.4 cm in size and moderate distal colonic diverticulosis (Figures 1-2). 

**Figure 3:**
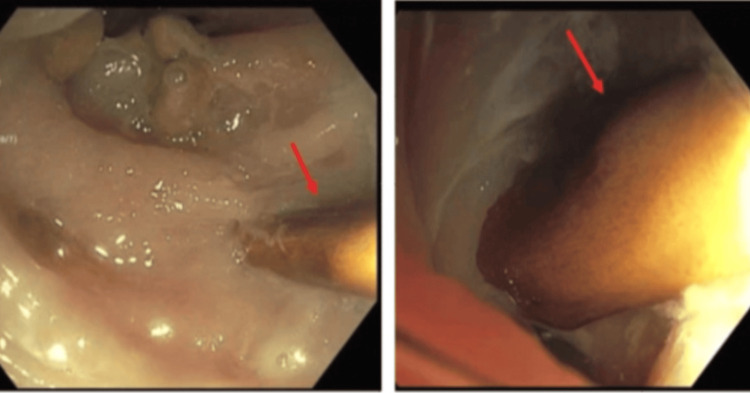
A sharp bony foreign body impacted in the sigmoid colon.

She was admitted for further management of retained sigmoid colon foreign body with perforation and abscess formation. Surgery, gastroenterology, and interventional radiology (IR) were consulted for the same. The patient underwent urgent flexible sigmoidoscopy using CO_2_ insufflation without bowel preparation. A 3-cm bony foreign body was found to be impacted in the sigmoid colon (Figure 3).

**Figure 4:**
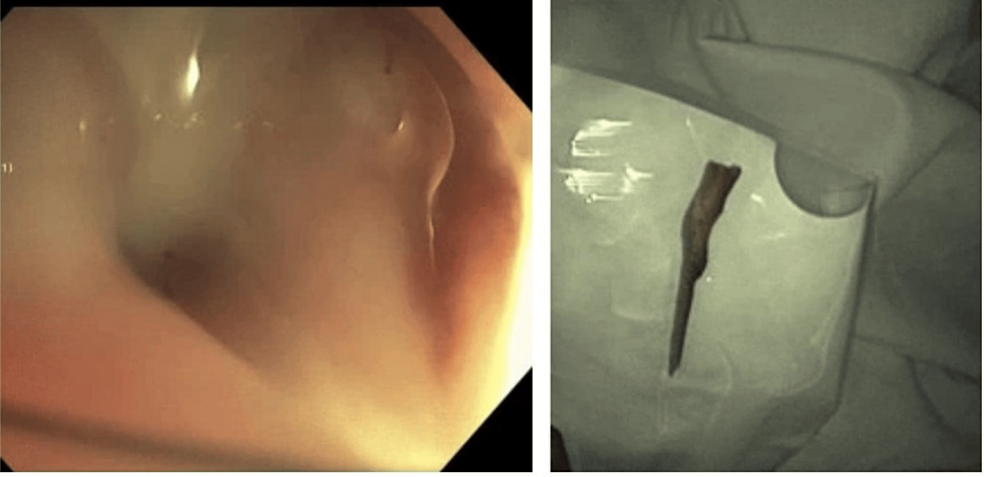
Successful removal of the foreign body.

It was successfully removed endoscopically with a snare. Many small- and large-mouthed diverticula were also found in the sigmoid colon. Pathology confirmed the foreign body as a bone (Figure 4).

**Figure 5:**
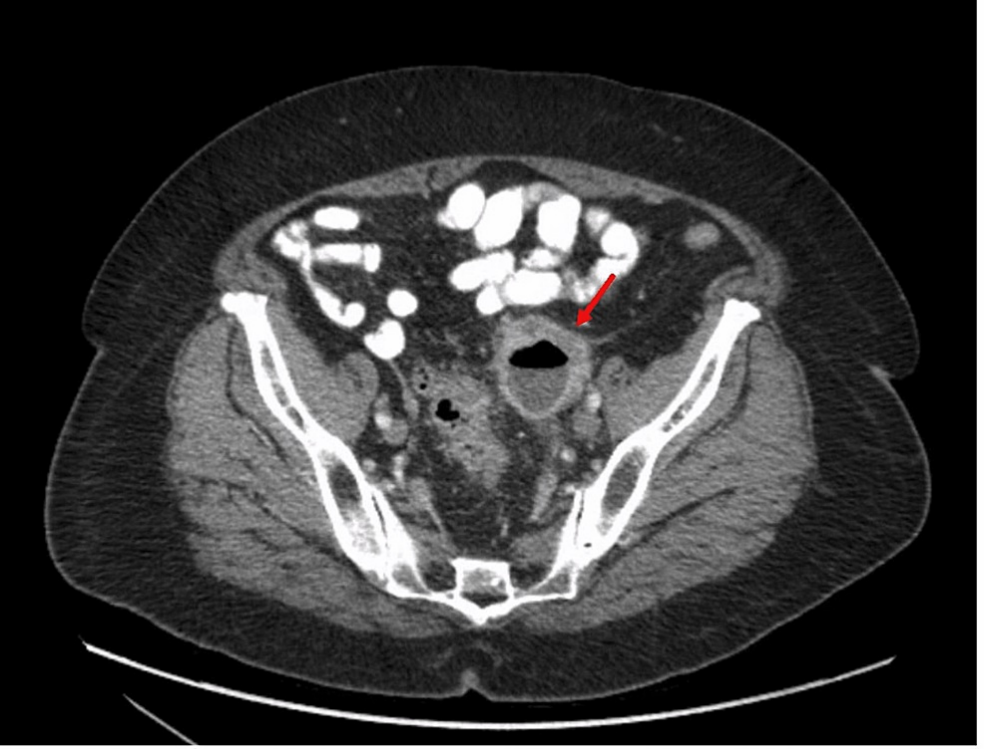
Follow-up CT abdomen pelvis with contrast image after three months showing persistent but slightly decreased abdominal abscess. CT, computed tomography

She underwent a repeat CT done five days later, which showed a stable abscess (Figure 5). 

The patient was planned for abscess drainage but had no safe window for drainage per IR evaluation. She was managed with broad-spectrum antibiotics (piperacillin-tazobactam) and clinically improved. She started on a clear liquid diet and later tolerated an advanced regular diet. The patient had one fever of (100.4 °F) during admission but remained afebrile and hemodynamically stable throughout the hospital stay. She was discharged on two weeks of oral antibiotics (ciprofloxacin and metronidazole) with outpatient follow-up. 

Upon outpatient follow-up, the patient remained clinically stable and tolerated food well. CRP improved to 8.89 mg/L. A repeat CT scan of the abdomen was performed three months after the initial encounter as an outpatient, revealing a persistent abdominal abscess with only a slight decrease in size and an increase in the interval CRP level from 8.89 to 24.20 mg/L. The patient was continued back on oral antibiotics and was referred to IR for another trial of abscess drainage; however, it was not amenable to drainage by IR. The patient was discharged with two weeks of ciprofloxacin and flagyl. The abscess was resolved with oral antibiotics, and during a subsequent clinic follow-up, the patient showed improvement. 

## Discussion

A variety of foreign bodies can be accidentally or intentionally swallowed. In about 80% of the cases, foreign bodies pass through the GI tract spontaneously without causing any problems [4]. Younger patients are more likely to pass out the foreign body without any endoscopic or surgical intervention as compared to elderly patients [5]. A foreign body stuck in the upper GI tract is the third most common nonbiliary endoscopic emergency [6]. According to a review article reviewing data on 1,391 patients, fish bone is the most common type of foreign body, and in the majority (66.6%) of the patients, the foreign body was found in the esophagus [7]. In the lower GI tract, the frequency is 20.3% in the colon, 5.5% in the sigmoid, and 10.6% in the rectum. Elderly patients, patients with dentures, alcoholic patients, visually impaired patients, patients with dementia, patients with psychiatric disorders, and inmates are at a great risk of ingesting foreign bodies [8]. According to a retrospective study done, patients aged more than 60 years were 2.53 times more likely to have foreign bodies (*P* < 0.019) [9]. Our patient is a 72-year-old female with a past medical history only significant for hypertension. 

Foreign body ingestion can present with a variety of symptoms and complications. Most of the patients are asymptomatic or have mild symptoms. One of the common symptoms associated with chicken bone ingestion is nonspecific abdominal pain [10]. Impaction of the chicken bone in the sigmoid colon is rare, and only a limited number of cases have been reported [2]. X-ray and CT of the abdomen are the most common imaging modalities used in diagnosing foreign bodies in the GI tract. Small foreign bodies such as bones can be concealed by soft tissue mass, food bolus, or fluids and might not appear on X-ray [4]. CT scan is a more useful imaging modality in these patients and patients with radiopaque foreign bodies. Some studies have shown that three-dimensional CT can be used in patients in which traditional diagnostic imaging modalities were unable to identify the foreign body [10]. The use of oral contrast in detecting foreign bodies has not been recommended due to the increased risk of aspiration in these patients and because foreign bodies might become obscured by the contrast media [11]. The complication of a foreign body includes ulceration, bleeding, GI obstruction, fistula formation, abscess formation, perforation, and sepsis. Most foreign objects that are swallowed and make their way to the stomach usually pass through the digestive system without causing any issues. However, when complications arise, how we handle these ingested objects depends on factors like their size, shape, material, and where they are located in the body. In such cases, after conducting imaging tests, performing endoscopy is an essential and safe diagnostic procedure. Conversely, if complications like abscesses and fistulas occur, surgical intervention becomes necessary [11].

## Conclusions

In summary, foreign objects found within the GI tract can give rise to a range of complex challenges. These challenges include the possibility of causing problems like abscesses, blockages, and perforations, along with the introduction of diagnostic difficulties, particularly when dealing with individuals who have underlying health conditions. Addressing such cases involves adopting a well-rounded strategy. A notable portion of these objects can transit through the digestive system without external intervention, while a subset demands either endoscopic or surgical procedures for resolution. This case underscores the importance of considering foreign body impactions in cases of unexplained abdominal pain, even without a history of predisposing factors. It also highlights the efficacy of minimally invasive surgical techniques in managing such cases. Clinicians and surgeons should remain vigilant for similar presentations, and early intervention is crucial to prevent potential complications associated with foreign body ingestion in the GI tract. 
